# Satisfaction with clinical pathway implementation versus job performance of clinicians: empirical evidence on the mediating role of work engagement from public hospitals in Sichuan, China

**DOI:** 10.1186/s12913-024-10856-w

**Published:** 2024-03-16

**Authors:** Junlong Li, Lu Ao, Jay Pan

**Affiliations:** 1 HEOA Group, West China School of Public Health and West China Fourth Hospital, Sichuan University, Chengdu, China; 2 Sichuan Vocational College of Health and Rehabilitation, Zigong, China

**Keywords:** Satisfaction with clinical pathway implementation, Job performance, Work engagement, Clinicians, Mediation

## Abstract

**Background:**

The job performance of clinicians is a clear indicator of both hospital capacity and the level of hospital service. It plays a crucial role in maintaining the effectiveness and quality of medical care. Clinical pathways are a systematic method of quality improvement successfully recommended by broader healthcare systems. Since clinicians play a key role in implementing clinical pathways in public hospitals, this study aims to investigate the effect of the satisfaction of clinicians in public hospitals with clinical pathway implementation on their job performance.

**Methods:**

A cross-sectional study design was used. Questionnaires were administered online. A total of 794 clinicians completed the questionnaires in seven tertiary public hospitals in Sichuan Province, China, of which 723 were valid for analysis. Questionnaires contained questions on social demographic characteristics, satisfaction with clinical pathway implementation, work engagement, and job performance. Structural Equation Model (SEM) was used to test the hypotheses.

**Results:**

The satisfaction of clinicians in public hospitals with clinical pathway implementation was significantly positively correlated with work engagement (*r* = 0.570, *P* < 0.01) and job performance (*r* = 0.522, *P* < 0.01). A strong indirect effect of clinicians’ satisfaction with clinical pathway implementation on job performance mediated by work engagement was observed, and the value of this effect was 0.383 (boot 95%CI [0.323, 0.448]).

**Conclusion:**

The satisfaction of clinicians in public hospitals with clinical pathway implementation not only directly influences their job performance, but also indirectly affects it through the mediating variable of work engagement. Therefore, managers of public hospitals need to pay close attention to clinicians’ evaluation and perception of the clinical pathway implementation. This entails taking adequate measures, such as providing strong organizational support and creating a favorable environment for the clinical pathway implementation. Additionally, focusing on teamwork to increase clinicians’ satisfaction can further enhance job performance. Furthermore, managers should give higher priority to increasing employees’ work engagement to improve clinicians’ job performance.

## Introduction

Job performance is an important concept in human resource management, which is the output of an organization’s activities to obtain the desired quantity, quality and efficiency of product or service [1]. The job performance of clinicians is their performance in the management of therapeutic treatment modalities and services offered by the hospital unit, which is an important manifestation of the capability and level of hospital service and is essential for the quality of medical services [2, 3]. Currently, job performance is widely used to evaluate the competence of individual members of medical staff to make clinical diagnosis, administer treatment, and to assess their achievements and adherence to healthcare quality standards [4]. Research on the factors influencing the job performance of medical staff was done by both international and domestic scholars. While international studies highlight factors such as organizational support [5, 6], teamwork [7, 8], job satisfaction [9], work engagement, and role awareness [5, 10], domestic research tends to emphasize psychological capital and work ability, as well as organizational commitment [11–13]. While existing research has made significant strides, much of the relevant literature predominantly examines healthcare systems in developed countries, with a particular emphasis on nurses. Studies addressing healthcare in developing countries, especially concerning doctors, are comparatively underrepresented in the literature [14]. Therefore, this study centers on clinicians in China and aims to provide academics with empirical evidence pertaining to the experiences of doctors in developing countries.

A clinical pathway is an interdisciplinary and integrated management model for clinical diagnosis and treatment, which is a standardized service plan for patients developed in a hospital by a group of multidisciplinary professionals for a particular disease. Its aim is to standardize procedures performed by medical staff, improve the quality and efficacy of medical services, and provide patients with better treatment [15, 16]. Clinical pathways are now widely used in the global healthcare industry [17]. More than 80% of hospitals in the United States implemented them as early as in 2003, and most European countries are using them now [18]. By 2021, 91.3% of Chinese public hospitals at the secondary level and above had implemented clinical pathways [19]. Given that clinicians play a critical role in the implementation of clinical pathways, their satisfaction with this process has a significant impact on the application of clinical pathways and the effectiveness of medical quality management to a certain extent in Chinese public hospitals. Satisfaction among clinicians with the implementation of clinical pathways refers to their perceptions of organizational structure, assurance mechanisms, process operations, and outcome evaluations within the clinical pathway process. This satisfaction generally includes three dimensions: organizational support, process identification, and effect perception [20]. Li et al.‘s study demonstrated that the satisfaction of medical staff with clinical pathway implementation had a significant positive effect on the quality of healthcare services [21]. However, in reality, public hospital administrators pay more attention to patients’ attitudes and evaluation of the effects of implementing clinical pathways [22, 23], while the attitudes and evaluations of doctors in this regard are usually overlooked. Hence, to enhance the quality of clinical pathway management in public hospitals, it is especially necessary to investigate doctors’ satisfaction with the implementation of clinical pathways and its impact on their work performance.

At present, there have been few studies exploring clinicians’ satisfaction with clinical pathway implementation [24], and even fewer examining the potential correlation between satisfaction and job performance. Recognizing the importance of this issue, the present study was designed and carried out to explore the relationship between satisfaction with clinical pathway implementation and job performance among Chinese clinicians.

## Methods

### Study framework and hypotheses

Previous studies have shown that employees’ job satisfaction is an important factor influencing job performance [25, 26], but the mechanisms by which job satisfaction affects job performance are not yet well understood [27]. Job satisfaction is an individual’s positive subjective evaluation or attitude towards various aspects of work, which is influenced by many factors such as the job itself, job challenges, compensation system, interpersonal relationships, working conditions, work motivation, and organizational environment [28–30]. Clinicians’ job satisfaction is their positive evaluation or attitude towards various aspects of medical work in hospitals. Since the recent medical reform in China implements clinical pathways, they have become a substantial part of clinicians’ work which consists mainly of diagnosing and administering treatment. By the end of 2021, more than 1000 diseases had been included in clinical pathway management in China [20]. Therefore, clinicians’ satisfaction with clinical pathway implementation seems crucial in deriving job satisfaction.

Previous research points out that job satisfaction should be studied along with work engagement, as both concepts relate to individual motivation [31]. Work engagement is defined as a positive, affective-motivational state of work-related well-being, characterized by vitality, dedication and focus [32, 33]. According to the job demand-resource model, job demands such as high workload, interpersonal conflicts, and unfavorable work environment may lead to dissatisfaction and burnout, and negatively affect the employee’s performance [34]. Job satisfaction generally includes satisfaction with nine aspects, i.e., pay, promotion opportunities, fringe benefits, rewards, supervision, interpersonal relationships, nature of work, communication, and working conditions [35]. The above nine aspects are on par with the satisfaction level of employees’ demands regarding their work environment. Consequently, high job satisfaction represents a high level of satisfaction with the work environment and other work demands, which can effectively motivate employees to work, and enhance their work engagement [36]. For example, to support the implementation of clinical pathways, clinicians need a robust electronic pathways system. A comprehensive electronic system better fulfills the conditions necessary for clinicians’ work, resulting in smoother workflow [37–39]. Consequently, the more motivated clinicians are to implement the clinical pathway, the degree of their engagement increases [37–39]. At the same time, employees with high work engagement have a strong sense of job identity and perform well at work [40].

In summary, we propose the use of the following hypotheses to determine the effect of satisfaction with clinical pathway implementation on job performance among clinicians in Chinese public hospitals.

#### Hypothesis 1

Clinicians’ satisfaction with clinical pathway implementation can positively influence job performance in public hospitals.

#### Hypothesis 2

Clinicians’ Satisfaction with clinical pathway implementation can positively influence work engagement in public hospitals.

#### Hypothesis 3

Work engagement plays a mediating role in the relationship between satisfaction with clinical pathway implementation and job performance among clinicians in public hospitals.

### Study design and participants

A cross-sectional design was used to conduct a questionnaire survey of clinicians in seven tertiary public hospitals in Sichuan. Inclusion criteria: (1) being a licensed clinician; (2) working for more than 1 year; (3) participating in clinical pathway implementation and management. Exclusion criteria: physicians in hospital training, advanced training and internship. Benter and Chou indicated that the sample size should ideally be 10 times the number of items in the analysis [41]. For this study, a total of 61 items were included, comprising 21 items related to satisfaction with clinical pathway implementation, 16 on work engagement, 16 on job performance, and 8 covariates. Thus, the minimum sample size of 610 was considered sufficient to achieve good statistical power. Finally, a total of 794 clinicians completed the questionnaires, of which 723 evaluable questionnaires were recovered, representing an efficiency rate of 91.06%.

### Data collection

Due to the COVID-19 pandemic restrictions and protocols, a field investigation was not conducted for this study. Data were collected using an online questionnaire tool, the Questionnaire Star website. The questionnaire link and QR code for the survey were distributed to clinicians working in various clinical departments with the assistance of the hospitals’ medical departments. The questionnaire consisted of a demographic information survey and three Chinese versions of maturation scales that had been validated in previous studies. Clinicians voluntarily participated in the survey after providing informed consent. The questionnaire was devised using appropriate Chinese language for clinicians, both in the questions and options, to increase the participation rates and improve the overall quality of the survey [42]. At the end of the survey, the questionnaire data were carefully reviewed and organized to remove incomplete questionnaires and outliers, to improve data representativeness.

### Measurements

#### Demographic characteristics

The demographic part of the questionnaire collected information on participants’ age, gender, marital status, education, income, professional title, length of service, and establishment status.

#### Satisfaction with clinical pathway implementation

This study adopted Satisfaction with Clinical Pathway Implementation Scale (SCPIS) developed by Li et al. [20] to assess clinicians’ satisfaction with clinical pathway implementation. The 21-item self-rating scale explored three dimensions: organizational support, process identity and perceived effectiveness. The relevant items in the questionnaire were structured in a Likert five-point format with 1 ~ 5 representing “very dissatisfied,” “slightly dissatisfied,” “uncertain,” “slightly satisfied,” and “very satisfied”, respectively. The items were scored positively, with higher scores indicating higher satisfaction levels. The minimum and maximum scores obtained from this scale were 21 and 105, respectively.

#### Work engagement

This study used the Work Engagement Scale (WES) revised by Li et al. [43] to assess clinicians’ work engagement. The 16-item self-rating scale explored three dimensions: vitality, dedication and concentration. The relevant items in the questionnaire were structured in a Likert seven-point scale ranging from “never” to “always”, and scores from 0 to 6, respectively. The items were scored positively, with higher scores indicating greater work engagement. The minimum and maximum scores obtained from this scale were 0 and 96, respectively.

#### Job performance

The Job Performance Scale (JPS) developed by Van Scotter et al. [44] was adopted for the study. The self-rating scale consists of 16 items, including three dimensions: work dedication, task performance and interpersonal facilitation. The scale used a Likert five-point format, with scores ranging from “very low” to “very high”, scored 1–5, respectively. The items were scored positively, with higher scores indicating greater job performance. The minimum and maximum scores obtained from this scale were 16 and 80, respectively.

### Data analyses

IBM SPSS 26.0 and AMOS 25.0 were used to enter and analyze the data. *P* < 0.05 (two-tailed) was used to indicate statistical significance. Categorical data were described as frequency and constituent ratio (%), and total and item scores on each scale were represented as means (± standard deviations). Cronbach’s coefficient alpha and confirmatory factor analysis were used to assess the validity and reliability of the questionnaire. Single factor analyses of job performance were conducted using the independent-sample t-test and one-way ANOVA. Pearson correlation analysis was used to analyze the correlation between each variable. Structural Equation Model (SEM), using maximum likelihood estimation, was conducted to determine the influence of satisfaction with clinical pathway implementation and work engagement on job performance [45]. To control for potential confounding variables, the statistically significant variables in the univariate analysis (gender and marital status) were included as control variables in the regression models [46, 47]. The fit indices of the models were evaluated using the following parameters: CMIN/df ≤ 5, root-mean-square error of approximation (RMSEA) ≤ 0.1, normed-fit index(NFI) ≥ 0.9, relative fit index (RFI) ≥ 0.9, comparative-fit-index (CFI) ≥ 0.9, goodness-of-fit index (GFI) ≥ 0.9 and parsimonious normal fit index (PNFI) ≥ 0.5 [48–52].

### Patient and public involvement

Patients or the public were not involved in the design, conduct, reporting, or dissemination plans of this research.

## Results

### Reliability and validity

The results of the reliability and validity analysis are shown in Table 1. Cronbach’s alpha coefficients for all three scales and their dimensions were higher than 0.8. The composite reliability (CR) values of each dimension of the three scales were higher than 0.8. The average variance extracted (AVE) values for each dimension of the three scales were higher than 0.5. These results support the validity and reliability of the research instruments.

**Table 1:** Validity and reliability testing of the scales

Scales	Dimension	Cronbach’s α coefficient	AVE	CR
SCPIS		0.973		
	Organizational support	0.956	0.701	0.955
	Process identity	0.920	0.747	0.922
	Perceived effectiveness	0.965	0.778	0.965
WES		0.961		
	Vitality	0.887	0.669	0.922
	Dedication	0.955	0.813	0.956
	Concentration	0.906	0.643	0.900
JPS		0.962		
	Work dedication	0.899	0.577	0.890
	Task performance	0.924	0.709	0.924
	Interpersonal facilitation	0.935	0.744	0.936

### Participant characteristics

Table 2 shows descriptive characteristics of the participants (*n* = 723). The participants included 393 males (54.36%) and 330 females (45.64%). The average age of participants was 35.37 ± 8.26 years.

**Table 2:** The demographic characteristics of participants (*n* = 723)

Variable	*N*(%)	Job performance		
		M ± SD	t/F	*P*
**Gender**			2.064	0.039
Male	393 (54.36)	4.506 ± 0.538		
Female	330 (45.64)	4.423 ± 0.541		
**Age(yrs old)**			2.218	0.085
20∼30	246 (34.02)	4.408 ± 0.570		
31∼40	325 (44.95)	4.475 ± 0.520		
41∼50	109 (15.08)	4.554 ± 0.499		
≥ 51	43 (5.95)	4.539 ± 0.596		
**Marital status**			-2.160	0.031
Unmarried	174 (24.07)	4.391 ± 0.560		
Married	549 (75.93)	4.492 ± 0.533		
**Education**			0.444	0.721
Junior college and below	39 (5.39)	4.463 ± 0.595		
Bachelor	483 (66.80)	4.478 ± 0.547		
Master	179 (24.76)	4.433 ± 0.533		
PhD	22 (3.04)	4.540 ± 0.324		
**Work income(yuan)**			0.489	0.744
<2000	13 (1.80)	4.298 ± 0.681		
2001∼5000	136 (18.81)	4.502 ± 0.578		
5001∼8000	293 (40.53)	4.459 ± 0.568		
8001∼10000	183 (25.31)	4.462 ± 0.465		
>10,000	98 (13.55)	4.480 ± 0.520		
**Professional title**			1.735	0.140
None	51 (7.05)	4.441 ± 0.546		
Primary	207 (28.63)	4.401 ± 0.577		
Intermediate	249 (34.44)	4.518 ± 0.488		
Associate chief physician	186 (25.73)	4.499 ± 0.545		
Chief physician	30 (4.15)	4.373 ± 0.627		
**Work years**			1.379	0.239
<5	197 (27.25)	4.421 ± 0.545		
5∼9	196 (27.11)	4.435 ± 0.553		
10∼19	200 (27.66)	4.503 ± 0.518		
20∼29	97 (13.42)	4.519 ± 0.561		
≥ 30	33 (4.56)	4.587 ± 0.496		
**Establishment personnel or not**			1.307	0.192
Yes	586 (81.05)	4.481 ± 0.532		
No	137 (18.95)	4.414 ± 0.576		

### Single factor analysis

The results are shown in Table 2. Statistically significant differences were found in job performance based on gender (*P* < 0.05) and marital status (*P* < 0.05). However, differences in job performance based on age, educational status, work income, professional titles, work years, and establishment personnel status were not statistically significant (*P* > 0.05).

### Correlation analysis

According to the correlation analysis (Table 3), the results showed that satisfaction with clinical pathway implementation was significantly positively correlated to work engagement (*r* = 0.570, *p* < 0.01) and job performance (*r* = 0.522, *p* < 0.01). Besides, work engagement was significantly positively correlated to job performance (*r* = 707, *p* < 0.01).

**Table 3:** The correlations between satisfaction with clinical pathway implementation, work engagement and job performance

Variables	Satisfaction with clinical pathway implementation	Work engagement	Job performance
Satisfaction with clinical pathway implementation	1		
Work engagement	0.570^**^, 95%CI (0.503, 0.637)	1	
Job performance	0.522^**^, 95%CI (0.457, 0.588)	0.707^**^, 95%CI (0.664, 0.747)	1

^**^P<0.01

### Mediation effect of work engagement

**Fig. 1:**
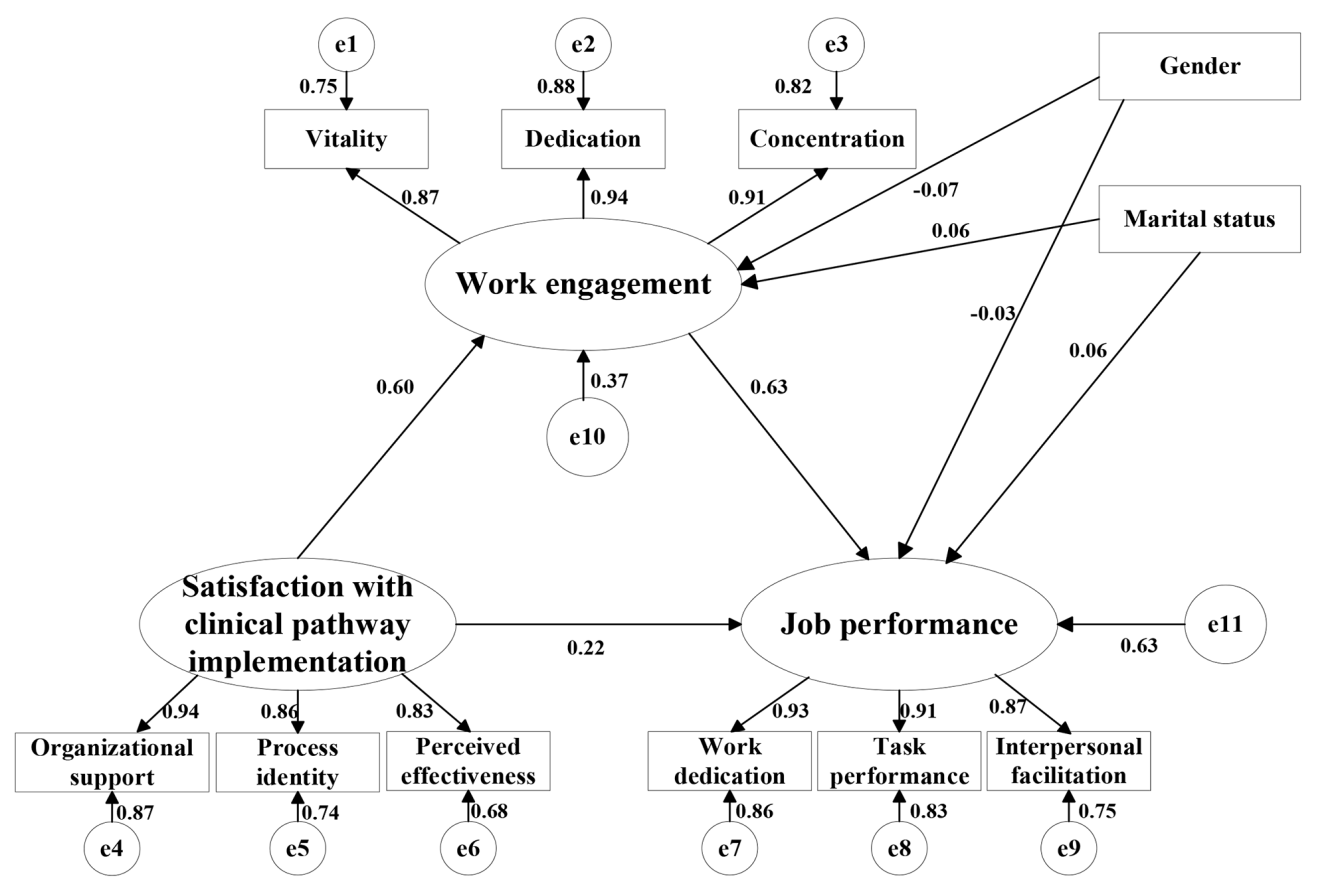
The mediation model

The study constructed a path diagram of a structural equation model of the satisfaction with clinical pathway implementation of clinicians in public hospitals as the independent variable work engagement as the mediating variable, and job performance as the dependent variable (Fig. 1). The model fitness fit indicators are shown in Table 4. The results showed that the model fit indices GFI > 0.9, NFI > 0.9, RFI > 0.9, CFI > 0.9, PNFI > 0.5 and RMSEA < 0.1 all met the reference standard, while CMIN/df > 5, was slightly higher than the reference standard. According to Wen et al., the CMIN/df value is affected by the sample size, and when the sample size is large, it cannot be used as a criterion to assess whether the model fits the data [53]. This study included a large sample size, of 723, and therefore, CMIN/df can be excluded as one of the model’s fit test indices. Consequently, the model can be considered as fitting well the data.

**Table 4:** The fit indices of the model

Parameters	CMIN/df	GFI	NFI	RFI	CFI	PNFI	RMSEA
Reference standards	≤ 5	>0.9	>0.9	>0.9	>0.9	>0.5	<0.1
Model fit indices values	6.670	0.934	0.957	0.940	0.963	0.679	0.089

Table 5 shows the results of the mediation effect analysis. As shown by the bias-corrected bootstrap, the boot 95% CI did not contain 0, indicating that the existence of the mediation effect was confirmed. Meanwhile, satisfaction with clinical pathway implementation had a significant positive effect on work engagement (*P* < 0.001), work engagement significantly positively predicted job performance (*P* < 0.001), and the direct effect of satisfaction with clinical pathway implementation on job performance was significant (*P* < 0.001), indicating that work engagement had partial mediation effects between satisfaction with clinical pathway implementation and job performance. The value of this standardized mediation effect was 0.383 (boot 95%CI [0.323, 0.448]).

**Table 5:** The mediating effects of work engagement

Path	Standardized coefficient	SE	CR	*P*
Satisfaction with clinical pathway implementation → Work engagement	0.604	0.032	15.848	0.000
Work engagement → Job performance	0.634	0.017	15.946	0.000
Satisfaction with clinical pathway implementation → Job performance	0.217	0.013	5.994	0.000
**Standardized mediation effect value**	**SE**	**Boot 95%CI**		***P***
0.383	0.032	0.323	0.448	0.000

## Discussion

The results of the correlation analysis showed a significant correlation between satisfaction with clinical pathway implementation and job performance among clinicians in public hospitals, and that satisfaction with clinical pathway implementation had a positive effect on job performance, indicating that Hypothesis 1 has been confirmed. These results are consistent with findings from numerous studies examining the interrelation between job satisfaction and job performance. Specifically, Ekingen’s study on nurses [9], Hou et al.’s study on residents [54], and Liu et al.’s study on primary healthcare workers [55] provide concrete evidence of this relationship. While other studies focusing on the relationship between satisfaction with clinical pathway implementation and job performance have not been retrieved, the results of this study can be corroborated with the findings of Askari et al. Their research showed that clinical pathway software improved healthcare workers’ satisfaction and job performance [56]. According to Li et al., clinicians perceived their own satisfaction with clinical pathway implementation in three dimensions: satisfaction with organizational support, process identity, and perceived effectiveness during clinical pathway implementation [20]. Related studies have shown that a high level of organizational support leads to the perception of reciprocity [57, 58]. When clinicians feel that the organization is willing and able to support them practically over the course of the implementation process, they, in return, will consciously engage in the behaviors expected by the hospital authorities, displaying more positive work attitudes and behaviors, thereby improving their job performance. As a standardized diagnosis and treatment mode, the standardized diagnosis and treatment implemented by a given clinical pathway are based on the guidelines developed by clinicians. As a result, effective cooperation within the department, also between the medical and nursing staff has a crucial role in implementing this clinical pathway. If clinicians show a low level of satisfaction with the programs and operating mechanisms during the implementation of a clinical pathway, it indicates that the implementation is challenging for them. Such a perception will inevitably reduce the efficiency of their work and negatively affect job performance. According to the social exchange theory and the principle of reciprocity [59], when an individual perceives that their effort is not proportional to the reward, this individual’s motivation to work will decrease, thereby reducing their job performance. A clinical pathway, as an important means to standardize clinicians’ responsibilities regarding giving diagnosis and administering treatment and to reduce patients’ medical costs, decreases, to a certain extent, the clinicians’ autonomy. Benefits from a clinical pathway for clinicians may include comparatively greater work efficiency, economic returns, quality of medical services or doctor-patient relationship after the implementation. If the clinicians’ perception of the benefits of the clinical pathway does not outweigh the effort required to implement it, their motivation and enthusiasm will decrease, and thereby their job performance. As a result when the clinicians’ perception of the clinicians’ a high level of satisfaction with clinical pathway implementation has, therefore, a positive effect on the job performance of clinicians in public hospitals.

Our findings show that work engagement has a partial mediating effect on the relationship between clinicians’ satisfaction with clinical pathway implementation and job performance in public hospitals. Namely, clinicians’ satisfaction with clinical pathway implementation not only directly, but also indirectly affects the job performance through the engagement in work, indicating that Hypothesis 2 and Hypothesis 3 have been confirmed. These hypotheses are backed by evidence from multiple studies. For instance, González-Gancedo et al. found a significant positive correlation between job satisfaction and work engagement among nurses [60]. Additionally, Bernales-Turpo et al. [61] and Zhang et al. [62] demonstrated in their research on medical staff that work engagement can function as a mediating variable for job performance and its predictors. Job satisfaction influences work engagement, which in turn is associated with higher job performance [63]. According to the job-demand resource theory, the factors affecting work engagement include both work demand and work resources, and if an individual’s work resources are continuously depleted and not replenished in a timely manner, their motivation for work and work engagement will be reduced [34, 64, 65]. Clinicians’ satisfaction with clinical pathway implementation is essentially their perception of organizational support, operating mechanisms, teamwork, and work effects during the clinical pathway implementation, which is an important component of clinicians’ work resources [66]. Such as when clinicians are unsatisfied with the implementation of clinical pathway, which means that benefits from the implementation of clinical pathway are smaller than the continuous input, their work motivation will decrease, which in turn will reduce their work engagement (i.e. they will devote less energy, focus and dedication to their work) [67]. Eventually, this will result in a reduction of in clinicians’ effectiveness and job performance. Taking into consideration the above, high levels of satisfaction of clinicians can help them engage more in work during implementing clinical pathways, which in turn will increase their job performance and ensure high standards of medical care.

### Limitations and recommendations

There are some limitations in this study that need to be noted for improvement in future research. First, this study is cross-sectional design. The data comes only from one point in time, which does not reflect the change of relevant variables over time nor causal relationship. Second, the sample of this study was only from one province in western China, which decreases the representativeness of the sample. Third, the data for the study came from participants’ self-reports that may be affected by social desirability bias. Lastly, our analysis focused solely on the overall relationship between clinicians’ satisfaction with clinical pathway implementation and job performance. In this study, we did not consider that there may be differences in clinicians’ satisfaction with clinical pathway implementation and job performance,.in the face of different pathway types and pathway implementation time. Acknowledging these four limitations, future studies may consider the following: (a) adopting a longitudinal design to test the causal relationship between the study variables; (b) using a larger sample to improve the representativeness of the data; (c) using more objective indicators to reduce data bias; and (d) taking into account different types of pathways and different implementation times to make the study more in-depth.

## Conclusion

To the best of our knowledge, this is the first study to offer empirical evidence on the impact of satisfaction of clinicians in public hospitals with clinical pathway implementation on their job performance. We confirmed that clinicians’ satisfaction with clinical pathway implementation in public hospitals has a direct positive effect on job performance, and also indirectly affects job performance through the mediating role of work engagement. Our findings provide evidence from a Chinese province for future research into relevant topics. The study suggests that the satisfaction among clinicians derived from effective clinical pathway implementation considerably improves their job performance. Therefore, public hospital administrators should take appropriate measures to improve the level of clinicians’ satisfaction with clinical pathway implementation in order to improve the work performance of this group as well as the quality of medical care.

## Data Availability

The datasets used and/or analysed during the current study are available from the corresponding author on reasonable request.
